# Transcription Factors From *Haematococcus pluvialis* Involved in the Regulation of Astaxanthin Biosynthesis Under High Light-Sodium Acetate Stress

**DOI:** 10.3389/fbioe.2021.650178

**Published:** 2021-10-25

**Authors:** Chaogang Wang, Kunpeng Wang, Jingjing Ning, Qiulan Luo, Yi Yang, Danqiong Huang, Hui Li

**Affiliations:** ^1^Shenzhen Key Laboratory of Marine Bioresource and Eco-Environmental Science, College of Life Sciences and Oceanography, Shenzhen University, Shenzhen, China; ^2^Guangdong Technology Research Center for Marine Algal Bioengineering, College of Life Sciences and Oceanography, Shenzhen University, Shenzhen, China; ^3^School of Life Science and Food Engineering, Hanshan Normal University, Chaozhou, China; ^4^Department of Biochemistry and Molecular Biology, Health Sciences Center of Shenzhen University, Shenzhen, China

**Keywords:** *Haematococcus pluvialis*, transcription factor, astaxanthin biosynthesis, regulatory network, high light-sodium acetate stress

## Abstract

The microalgae *Haematococcus pluvialis* attracts attention for its ability to accumulate astaxanthin up to its 4% dry weight under stress conditions, such as high light, salt stress, and nitrogen starvation. Previous researches indicated that the regulation of astaxanthin synthesis might happen at the transcriptional level. However, the transcription regulatory mechanism of astaxanthin synthesis is still unknown in *H. pluvialis*. Lacking studies on transcription factors (TFs) further hindered from discovering this mechanism. Hence, the transcriptome analysis of *H. pluvialis* under the high light-sodium acetate stress for 1.5 h was performed in this study, aiming to discover TFs and the regulation on astaxanthin synthesis. In total, 83,869 unigenes were obtained and annotated based on seven databases, including NR, NT, Kyoto Encyclopedia of Genes and Genomes Orthology, SwissProt, Pfam, Eukaryotic Orthologous Groups, and Gene Ontology. Moreover, 476 TFs belonging to 52 families were annotated by blasting against the PlantTFDB database. By comparing with the control group, 4,367 differentially expressed genes composing of 2,050 upregulated unigenes and 2,317 downregulated unigenes were identified. Most of them were involved in metabolic process, catalytic activity, single-organism process, single-organism cellular process, and single-organism metabolic process. Among them, 28 upregulated TFs and 41 downregulated TFs belonging to 27 TF families were found. The transcription analysis showed that TFs had different transcription modules responding to the high light and sodium acetate stress. Interestingly, six TFs belonging to MYB, MYB_related, NF-YC, Nin-like, and C3H families were found to be involved in the transcription regulation of 27 astaxanthin synthesis-related genes according to the regulatory network. Moreover, these TFs might affect astaxanthin synthesis by directly regulating *CrtO*, showing that *CrtO* was the hub gene in astaxanthin synthesis. The present study provided new insight into a global view of TFs and their correlations to astaxanthin synthesis in *H. pluvialis*.

## Introduction

*Haematococcus pluvialis*, which belonged to Chlorophata, Chlorophyceae, Volvocales, and Haematococcaceae, was paid close attention to its ability to accumulate astaxanthin up to its 4% cellular dry weight. Under favorite environment, *Haematococcus* cells is a green cell swimming with its flagellum, while generating a thick cell wall and produce astaxanthin under stress conditions, such as high light, salt stress, and nitrogen starvation (Lemoine and Schoefs, 2010; Shah et al., 2016; Zhao et al., 2020). However, the regulatory mechanism of astaxanthin biosynthesis causing by abiotic stresses is still unknown.

Previous researches showed that *H. pluvialis* accumulated huge astaxanthin under high light and sodium acetate stresses, with the observation that the transcription activity of genes related to astaxanthin biosynthesis increased quickly (Huang et al., 2006; Raman et al., 2008). As well known, light is the most important contributor to microalgae growth. Normally, the light intensity suitable for growth is 25–50 50 μmol m^–2^ s^–1,^ and it would cause the accumulation of astaxanthin when more than 100 100 μmol m^–2^ s^–1^ depending on the light tolerance of different *H. pluvialis* strains (Steinbrenner and Linden, 2001; Steinbrenner and Linden, 2003; Su et al., 2014). The sodium acetate was also noticed because it helped the growth of *H. pluvialis* and improved the cell density. Specially, sodium acetate has been reported to contribute to the accumulation of astaxanthin under high light (He et al., 2018; Jeon et al., 2006). The transcription levels of astaxanthin synthesis-related genes were improved to accelerate the astaxanthin synthesis under environmental stresses. According to previous reports, astaxanthin biosynthesis in *H. pluvialis* started from isopentenyl pyrophosphate, gradually formed phytoene, β-carotene, and then finally produced astaxanthin by β-carotene hydroxylase and β-carotene ketolase (Lemoine and Schoefs, 2010; Uri et al., 2019). Increasing astaxanthin yield under stresses, astaxanthin biosynthesis-related enzymes such as phytoene synthase (PSY), phytoene desaturase (PDS), zeta-carotene desaturase (ZDS), beta-carotene ketolase (BKT), and beta-carotene hydroxylase (crtR-B) transcribed at a high level within 24 h, suggesting that the regulation of astaxanthin synthesis might happen at the transcriptional level (Steinbrenner and Linden, 2003; Lu et al., 2010; Gao et al., 2012a; Wen et al., 2015).

Responses to stresses in the plant could be achieved by the complex signal transduction, including the expression of transcription factors (TFs), promoter binding, and the regulation of functional genes. Hence, transcriptional regulation was closely related to the function of TFs and the *cis*-acting elements in the promoter. Till now, the research on the correlation between TFs and astaxanthin synthesis is still blank, although a lot of transcriptome data were published. The *cis*-acting elements in the *CrtO*, *BKT* promoter were obtained, including ABA, ABRE, C-repeat/DRE, G-box, MeJA-responsive element, and MBS (Meng et al., 2005; Gao et al., 2010; Wang et al., 2012). In addition, a little achievement was made when 59 TFs belonging to MYB, bHLH, bZIP, and C2H2 families were identified when treated with salicylic acid or jasmonic acid, suggesting that TFs might involve in hormone stress (Gao et al., 2015). A lot of research focused on astaxanthin biosynthesis and lipid biosynthesis but overlooked the function of TFs, leading to the failure of exposing the regulatory mechanism of astaxanthin biosynthesis.

In this study, *H. pluvialis* under high light-sodium acetate (HLS) stress was chosen to proceed with transcriptome analysis. Fortunately, many TFs were found at the early stage of HLS stress so that we could study the response patterns and the internal connection between TFs and astaxanthin biosynthesis-related genes. Totally, 83,869 unigenes were identified, among which 476 unigenes were annotated as TFs. After that, 69 differentially expressed transcription factors (DETFs) were obtained, and their transcription profiles of high light and sodium acetate stress responses were studied. Results revealed that their expression was changed along with different stresses. In addition, we finally constructed a TF–astaxanthin biosynthesis regulatory network obtaining six important TFs that might directly modulate key genes of astaxanthin biosynthesis, based on a combined analysis of DETFs, expression trends, and the analysis of the transcription factor binding site (TFBS). This is the first report to take a look inside the correlations between TFs and astaxanthin biosynthesis-related genes *via* gene expression trends and TFBS. Results in this study would not only help us understand the response of TFs to environmental stresses but also provide a new way to obtain key TFs involved in the modulation of astaxanthin biosynthesis.

## Materials and Methods

### Algal Strains and Culture Conditions

*Haematococcus pluvialis* strain 192.80 was purchased from Sammlung von Algenkulturen Göttingen Culture Collection of Algae. It was cultured in MIX medium, with a 12:12-h light/dark cycle at 22°C (Luo et al., 2017). The light intensity for normal culture was 25 μmol m^–2^ s^–1^ while 550 μmol m^–2^ s^–1^ for the high light stress (HL). The final concentration of sodium acetate was 45 μM for the sodium acetate stress (HS). The alga was cultured until the cell concentration reached 4–5 × 10^5^ cells/ml and then treated with HLS stress. *H. pluvialis* cells were treated with a light intensity of 550 μmol m^–2^ s^–1^ and 45-μM sodium acetate for HLS. Also, the *H. pluvialis* cells under a light intensity of 25 μmol m^–2^ s^–1^ were the control group.

### Microscopy Observation of Algal Cells

Algal cells were treated with HLS stress when the cell concentration reached 4–5 × 10^5^ cells/ml. After sampling at 0, 0.5, 1, 1.5, 2, 2.5, 3, 4, 5, 6, 7, 8, 9, 10, 11, and 12 h, the alga cells were observed using an Olympus BX61 microscope and an Olympus DP10 digital camera.

### Total RNA Extraction and Quality Control

Total RNA was extracted according to the modified RNA isolation methods (Luo et al., 2017). The RNA quality and quantity were checked by 1% agarose gel and spectrophotometer NanoDrop (BioPhotometer, Eppendorf, Hamburg, Germany).

### Complementary DNA Library Construction and Transcriptome Sequencing

The eukaryotic messenger RNA was enriched with Oligo (dT) magnetic beads and then added with a fragmentation buffer. Random hexamers were used to finish single-stranded complementary DNA (cDNA) synthesis, deoxyribonucleotide triphosphates, and DNA polymerase I, and RNase H was then added to synthesize the second-strand cDNA. AMPure XP beads purified those fragments. After amplification by polymerase chain reaction (PCR), the products were purified by AMPure XP beads to construct the final cDNA library. Qubit2.0 was used to dilute the cDNA concentration to 1.5 ng/μl. Then, the insert size was checked with Agilent 2100. After that, the effective concentration of the cDNA library was accurately quantified by the quantitative PCR method, making sure that the effective concentration of 2 nM was reached. Finally, transcriptome sequencing was performed by Illumina HiSeq^TM^ (Illumina, Santiago, CA, United States). The transcriptome data has been uploaded to the National Center for Biotechnology Information (NCBI) website (accession number: SRR6816386).

### Sequence Assembly and Gene Annotation

The assembled sequences were annotated with several protein databases, including NR (NCBI non-redundant protein sequences), NT (NCBI nucleotide sequences), Kyoto Encyclopedia of Genes and Genomes (KEGG) Ortholog (KO), SwissProt (a manually annotated and reviewed protein sequence database), Pfam (protein family), Eukaryotic Orthologous Groups (clusters of orthologous groups of proteins) (KOG), and Gene Ontology (GO). Cufflinks and HTSeq package in 2010 were used to calculate the fragments per kilobase of transcript, per million mapped reads value and read counts of each gene, respectively. Also, the differentially expressed genes (DEGs) were identified using the DEGSeq, with *p*-adjust < 0.05 and |log_2_FC| > = 1 setting as the threshold to indicate significant differential expression.

### Annotation and Classification of Transcription Factors

All the unigenes, including known genes and new genes, were blasted against plantTFDB^1^ database to obtain TF data. Those TFs were classified according to the information in plantTFDB.

### Validation of Gene Expression by Real-Time Quantitative Reverse Transcription Polymerase Chain Reaction

*Haematococcus pluvialis* cells were cultured until cell numbers reached 4–5 × 10^5^ cells/ml, then treated with a light intensity of 25 μmol m^–2^ s^–1^ as control, 550 μmol m^–2^ s^–1^ as high light (HL), 25 μmol m^–2^ s^–1^ plus 45-mM sodium acetate as sodium acetate stress (HS), and 550 μmol m^–2^ s^–1^ plus 45-mM sodium acetate as HLS stress. The real-time quantitative reverse transcription (qRT)-PCR was performed using an SYBR Premix Ex Taq^TM^ II Kit (TaKaRa, Tokyo, Japan) in a 20-μl reaction system containing 10-μl 2 × SYBR Premix Ex Taq^TM^ II (TaKaRa, Tokyo, Japan), 0.8 μl each of the forward and reverse primers, 2-μl cDNA, 0.4-μl 50 × ROX Reference Dye, and 6-μl double distilled water. Reaction steps followed the program: 95°C for 30 s, followed by 40 cycles of 95°C for 5 s, 55°C for 31 s, and reading the fluorescence signal. Each qRT-PCR reaction was performed with three biological replicates. β-actin of *H. pluvialis* was used as internal control. All primers were designed by the Primer Premier 5.0 and are listed in Supplementary Table 1. The relative transcription level was calculated using the 2^–ΔΔCT^ method.

### Construction of Transcription Factor–Astaxanthin Synthesis Regulatory Network

Obtaining the target genes of TFs and considering the relationship between TFs and their target genes, we could generate the network reflecting the regulation between TFs and their target genes. Firstly, DETFs should obtain as a dataset. Then, DEGs related to astaxanthin synthesis were collected as another dataset. According to the TFBS information in the promoter of astaxanthin synthesis-related genes, we could calculate the correlation between TFs and astaxanthin synthesis-related genes by searching the JASPAR database. Finally, the network between TFs and target genes was obtained.

### Statistical Analysis

All experiments were performed with biological triplicates from separate cultures. GraphPad Prism 5 was used for statistical analysis, and *P*-values of <0.05 were considered statistically significant.

## Results

### Microscopic Observation and Transcription Analysis of *H. pluvialis* Under High Light-Sodium Acetate Stress

**FIGURE 1:**
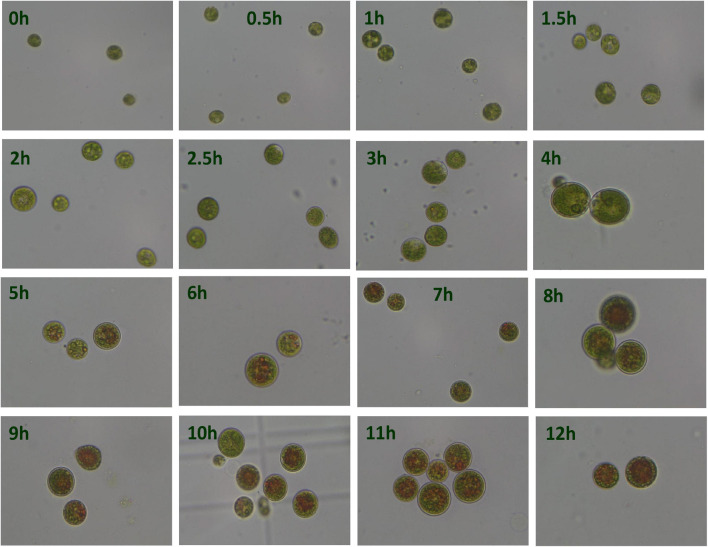
Microscopic observation of *Haematococcus pluvialis* under high light-sodium acetate (HLS) stress.

**FIGURE 2:**
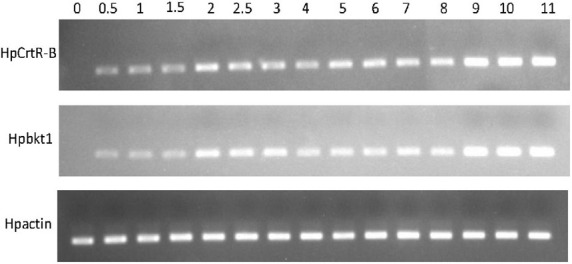
Expression pattern of *HpCrtR-B* and *Hpbkt1* responding to HLS stress.

Under the HLS condition, the content of astaxanthin in algal cells was increased with the time course. *H. pluvialis* cells were turned red after HLS stress for 4 h and then the red getting darker along with the extension time of stress treatment (Figure 1). The transcription of *HpCrtR-B* and *Hpbkt1* was observed under HLS stress for 0.5 h, suggesting that they could respond to stress quickly. Totally, the expression level of *HpCrtR-B* and *Hpbkt1* was kept increasing, with the exception that their expression was downregulated after stressing for 4–5 h (Figure 2). Therefore, samples after 1.5 h stress treatment were selected for transcriptome analysis.

### Transcriptome Sequencing and Assembly

**FIGURE 3:**
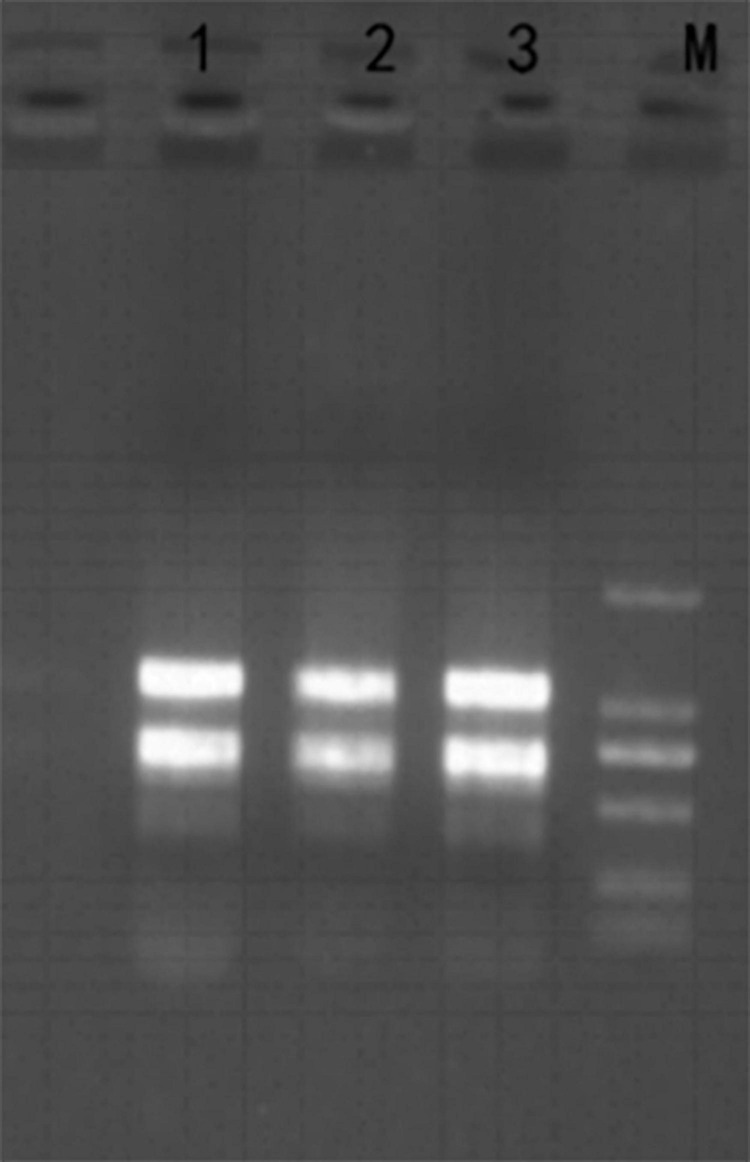
Total RNA from *H. pluvialis* 192.80. Lane M was DL2000 marker, and lanes 1–3 represent three separate samples as replicates.

Firstly, total RNA was extracted from *H. pluvialis* treated with HLS stress, and its quality was checked with Nanodrop and 1% agarose gel (Figure 3). The A260/280 and A260/230 were more than 2, and the RNA integrity number value was 7.0, showing that the quality of extraction RNA was great to conduct transcriptome sequencing.

Then, total RNA was reverse transcribed to cDNA and proceeded transcriptome sequencing. In total, the quality of Q20 was more than 94%, and the GC content was around 58.16% (Table 1). Here, 117,861 transcripts and 83,869 unigenes were obtained, with an average length of 615 and 747 bp, respectively. In unigenes, the N50 and N90 were 1,268 and 292 bp, respectively (Table 2).

**TABLE 1:** List of data output quality.

**Sample**	**Raw Reads**	**Clean Reads**	**CleanBases**	**Error(%)**	**Q20(%)**	**Q30(%)**	**GC Content(%)**
HLST1	50385816	47984828	6G	0.03	95.03	90.21	58.09
HLST2	52595368	50248474	6.28G	0.03	95.22	90.55	57.98
HLST3	53089534	50392078	6.3G	0.03	95.32	90.7	58.12
LLMT4	54231624	51614202	6.45G	0.03	95.17	90.38	58.36
LLMT5	62538310	59057482	7.38G	0.03	94.64	89.41	58.3
LLMT6	53201140	50665756	6.33G	0.03	95.06	90.21	58.12

*Samples of HLST1, HLST2, and HLST3 were three biological replicates treated with HLS stress for 1.5 h. Samples of LLMT4, LLMT5, and LLMT6 were three biological replicates cultured in normal conditions as controls.*

**TABLE 2:** Length distribution of transcripts and unigenes.

**Transcript length interval**	**200–500 bp**	**500–1,000 bp**	**1,000–2,000 bp**	**>2,000 bp**	**Total**
Number of transcripts	69,950	22,470	16,350	9,091	117,861
Number of Unigenes	57,664	13,813	7,968	4,424	83,869

### Gene Annotation

All unigenes were blasted against seven databases, including NR, NT, KO, SwissProt, PFAM, KOG, and GO. As a whole, there were 23,928 unigenes (28.53%) annotated in at least one database; 1,969 unigenes (2.34%) were annotated in all databases. Specifically, 18,177 unigenes (21.67%), 18,162 unigenes (21.65%), 13,464 unigenes (16.05%), 9,931 unigenes (11.84%), 7,154 unigenes (8.52%), 6,158 unigenes (7.34%), and 5,767 unigenes (6.87%) were annotated in PFAM, GO, NR, SwissProt, KOG, KO, and NT, respectively. In addition, *H. pluvialis* shared 32.6% homology with *Volvox carteri* and 25.9% homology with *Chlamydomonas reinhardtii* (Supplementary Figure 1). A great number of unigenes associated with gene transcription, signal transduction, and TF activity were found in GO, KOG, and KEEG analyses, suggesting that gene transcription was active and TFs might be involved in the response of HLS stress.

### Prediction and Classification of Transcription Factors

Transcription factors play a key role in a plant when responding to environmental stresses. However, few references were focused on TFs in *H. pluvialis* and hindered the research of transcriptional regulation to stresses. Hence, to understand their functions in transcriptional regulation, we predicted all the TFs in *H. pluvialis* by blasting against PlantTFDB. Totally, 476 TFs belonging to 52 families were predicted and annotated. According to the searching data, the top five families were C3H (8.6%), SET (8.6%), GNAT (7.98%), SNF2 (7.1%), and MYB (5.9%) (Table 3). It is well known that the C3H family is related to the regulation of light and photoperiod. In addition, TF families such as WRSKY, MYB, and AP2-EREBP are generally associated with abiotic stress responses in plants. Here, many TFs were found in *H. pluvialis* during an early stage of HLS stress, which might be related to transcription regulation to the stress response.

**TABLE 3:** List of transcription factors (TFs).

**Family name**	**Numbers**	**Family name**	**Numbers**	**Family name**	**Numbers**
ABI3VP1	1	DDT	1	PBF-2-like	1
AP2-EREBP	20	E2F-DP	2	PHD	21
ARID	3	FHA	14	PLATZ	2
ARR-B	1	G2-like	2	RB	1
bHLH	3	GNAT	38	Rcd1-like	1
BSD	3	HB	4	RWP-RK	13
bZIP	17	HMG	9	SBP	9
C2C2-co-like	1	HSF	2	SET	41
C2C2-Dof	1	IWS1	3	Sigma70-like	1
C2C2-GATA	14	Jumonji	16	SNF2	34
C2C2-YABBY	1	LIM	2	SOH1	2
C2H2	22	MBF1	2	SWI/SNF-BAF60b	1
C3H	41	MED6	2	SWI/SNF-SWI3	2
CCAAT	12	MED7	1	TAZ	6
Coactivator p15	2	mTERF	3	TIG	8
CPP	6	MYB	28	TRAF	9
CSD	8	Orphans	34	TUB	2
WRKY	3				

### Differentially Expressed Genes Under High Light-Sodium Acetate Stress

**FIGURE 4:**
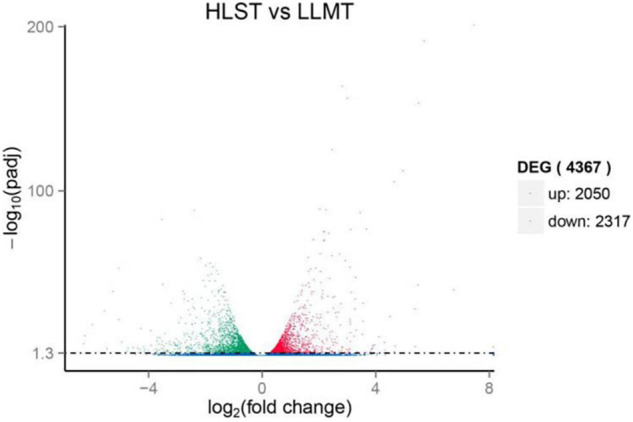
Expression analysis of differentially expressed genes (DEGs). Y-axis represents statistical significance of DEGs. X-axis represents change fold of gene expression. Red and green dots represent upregulated genes and downregulated genes, respectively. Blue dots represent genes with no significant difference in expression level.

To find out genes involving in early stress response, unigenes from the control group cultured under normal conditions were compared with that from the treatment group under HLS stress. As expected, results provided that they held 40,275 unigenes as common, whereas the control group had 12,803 different unigenes and the treatment group owned 10,724 different unigenes (Supplementary Figure 2). In addition, there were 4,367 DEGs composed of 2,050 upregulated unigenes and 2,317 downregulated unigenes (Figure 4).

The GO enrichment analysis showed that GO terms were significantly enriched in the treated group, indicating that the top five terms were metabolic process, catalytic activity, single-organism process, single-organism cellular process, and single-organism metabolic process (Supplementary Figure 3). Interestingly, 620 unigenes were involved in the catalytic function, and 12.6% unigenes were related to the binding process of iron and metal ions, showing that enzymatic catalysis and metal ions might play important roles during the early stage of HLS stress. The KEEG enrichment showed that the upregulated DEGs were significantly enriched in KEGG terms. The top five terms were “Protein processing in endoplasmic reticulum,” “Proteasome,” “Non-alcoholic fatty liver disease,” “Plant-pathogen interaction,” and “Tuberculosis.” As expected, 11 upregulated DEGs were involved in carotenoid biosynthesis, including LUT1, lcyB, crtL1, crtY, ZDS, crtQ, PDS, crtP, crtZ, crtB, and Z-ISO. Moreover, the downregulated DEGs were significantly enriched in KEGG terms of “Purine metabolism,” “Pyrimidine metabolism,” and “Biosynthesis of amino acids.”

### Obtainment of Differentially Expressed Transcription Factors

Although TFs played an important role in response to abiotic stresses, researches on transcriptome under stresses did not expose their functions in *H. pluvialis*. Among the 476 TFs, there were 28 upregulated TFs and 41 downregulated TFs when compared with the control group. The DETFs belonged to 27 TF families, such as SET (13%), GNAT (10%), and Orphans (10%) (Table 4). Among the upregulated DETFs, GNAT, and Orphans families owned the same numbers as four, whereas SET families had the most as eight among the downregulated DETFs. In addition, TFs from GNAT, MYB, Orphans, PHD, and bZIP families were found in both upregulated and downregulated TFs.

**TABLE 4:** Differentially expressed transcription factors (DETFs) and their classification.

	**TF family**	**Numbers**		**TF family**	**Numbers**
Down-regulated TFs	SET	8	Up-regulated TFs	GNAT	4
	SNF2	5		Orphans	4
	CCAAT	3		C2C2-GATA	2
	GNAT	3		CCAAT	2
	MYB	3		MYB	2
	Orphans	3		RWP-RK	2
	C2C2-GATA	2		TRAF	2
	C3H	2		bHLH	1
	Coactivator p15	2		bZIP	1
	PHD	2		C2C2-co-like	1
	AP2-EREBP	1		C2H2	1
	bZIP	1		CPP	1
	CSD	1		G2-like	1
	E2F-DP	1		HB	1
	MED7	1		MBF	1
	Rcd1-like	1		PHD	1
	Sigma7C0-like	1		SNF	1
	WRKY	1			
Total number		41			28

**FIGURE 5:**
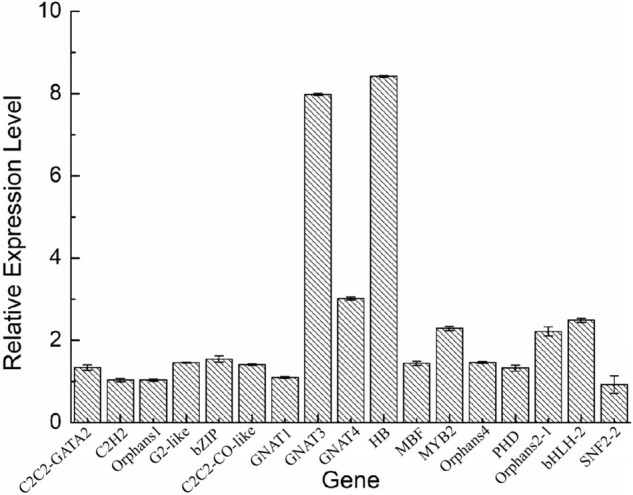
Analysis of upregulated differentially expressed transcription factors (DETFs) by qRT-PCR.

**FIGURE 6:**
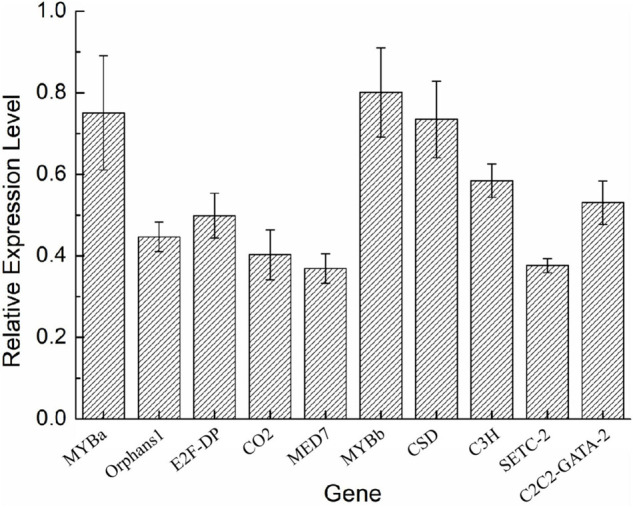
Analysis of downregulated DETFs by quantitative reverse transcription (qRT)-PCR.

To validate the expression of the DETFs, total RNA was extracted from the *H. pluvialis* under normal culture conditions and HLS stress. A total of 26 DETFs were randomly selected to verify their expression. Most of them were confirmed that the expression of TFs in transcriptome analysis under HLS stress was credible (Figures 5, 6). What attracted our attention was the expression of GNAT3 and HB, showing strong upregulation with more than eightfold, which was not previously reported before.

### Transcription Factors Expression Profiles Under High Light Stress and Sodium Acetate Stress

**FIGURE 7:**
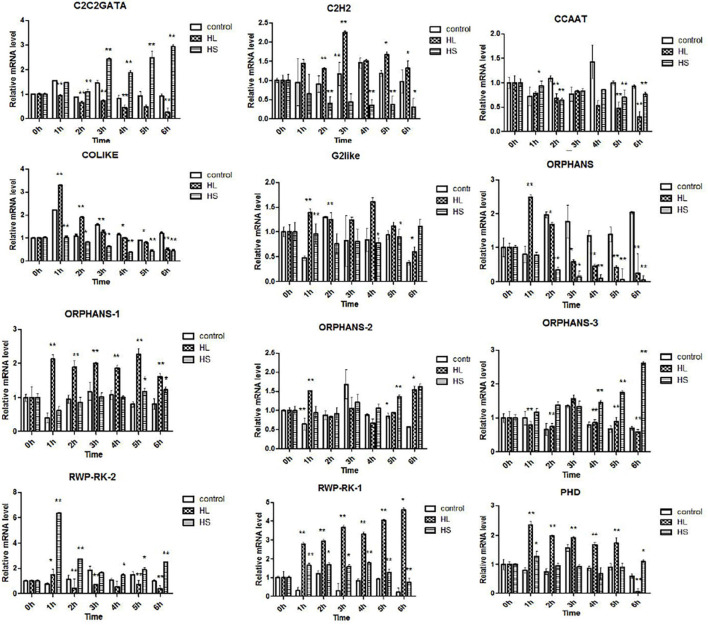
Transcription profiles of transcription factors (TFs) under high light and sodium acetate stress. Control represents control group, HL represents high light stress, and HS represents sodium acetate stress. *Indicates significant difference at level of 0.05, and **indicates significant difference at level of 0.01.

In this research, 476 TFs were found, and 69 DETFs belonging to different TF families were obtained. Among them, TFs related to stress response were chosen for further study, helping us understand their function. Therefore, 14 DETFs were selected to analyze their transcription profiles under HL and HS from 0 to 6 h, respectively. TFs showed different transcription activities under HL and HS, suggesting that they responded to stresses differentially, which might be due to their varied regulation pattern. In general, results showed that most of TFs were upregulated at the stress condition of HL or HS. TFs such as C2H2, COLIKE, G2like, ORPHANS-1, PHD, and RWP-RK-1 were sensitive to HL, and C2C2GATA, ORPHANS-3, and RWP-RK-2 were positive to HS. ORPHANS-2 could respond to both HL and HS. Interestingly, four TFs of ORPHANS families showed very different transcriptional profiles, in which ORPHANS-1 was positive to HL, ORPHANS-2 was normal sensitive to HL and HS, ORPHANS-3 was positive to HS, and the expression of ORPHANS was negative to HL and HS except that its expression increased 2.6-fold after HL at 1 h. The same appearance was found in RWP-RK families, in which RWP-RK-1 was positive to HL with a 4.2-fold increase, whereas RWP-RK-2 was positive to HS with a 6.5-fold increase (Figure 7). Those results implied that TFs could respond to different kinds of stresses, although they were from the same TF families.

### Expression of Key Genes Associated With Astaxanthin Synthesis

**FIGURE 8:**
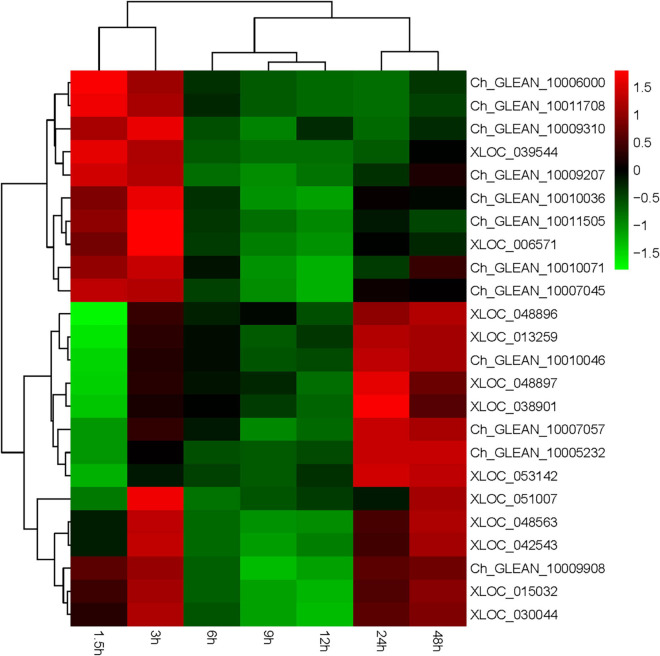
Expression of astaxanthin synthesis-related genes from 0 to 48 h.

*Haematococcus pluvialis* under HLS stress was sampled in 0, 1.5, 3, 6, 9, 12, 24, and 48 h and extracted total RNA to finish transcriptome analysis. Also, 24 key genes related to astaxanthin synthesis were found from transcriptome data. Totally, three *ipi*, four *ggps*, two *psy*, two *pds*, three *zds*, three *lcy*, four *bkt*, one *crtz*, and two *crtR-B* genes were found, and their expression trends showed three expression modes (Supplementary Table 2). The first mode included 13 genes (Ch_GLEAN_10009908, XLOC_ 015032, XLOC_030044, Ch_GLEAN_10009310, XLOC_03 954, Ch_GLEAN_10006000, Ch_GLEAN_10011708, Ch_GLE AN_10007045, Ch_GLEAN_10009207, Ch_GLEAN_10010071, Ch_GLEAN_10010036, Ch_GLEAN_10011505, and XLOC_ 006571), showing their highest transcription level that happened after stress at 1.5 or 3 h. Especially, the transcription of *crtR-B* (Ch_GLEAN_10006000) was increased to 24-fold. The second mode had 11 genes (XLOC_013259, Ch_GLEAN_10007057, XLOC_038901, XLOC_051007, XLOC_048563, XLOC_042543, XLOC_048896, XLOC_048897, Ch_GLEAN_10005232, Ch_GLEAN_10010046, and XLOC_053142), showing that their highest expression level was reached at 24 or 48 h. Among them, the expression of *BKT* (XLOC_038901) and *CRTZ* (Ch_GLEAN_10005232) were upregulated up to 19.6- and 16.07-fold at 24 h, respectively. Moreover, the expression of *IPI* (XLOC_051007) and *PSY* (XLOC_042543 and XLOC_048563) were significantly increased at both 3 and 48 h with similar levels (Figure 8 and Supplementary Table 3).

### Construction of Transcription Factor–Astaxanthin Synthesis Regulatory Network

**FIGURE 9:**
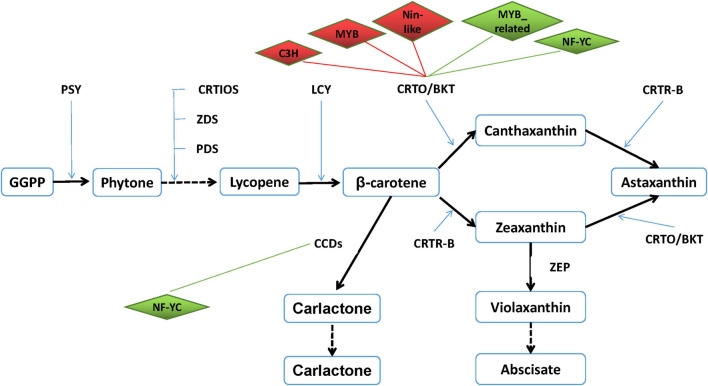
Transcription factor–astaxanthin synthesis regulatory network. Diamond represents transcription factor. Upregulated TF is marked in red, whereas downregulated TF is in green. Rectangle represents astaxanthin synthesis-related gene. Line in red means their relationship is positive, whereas line in green means their relationship is negative.

It is well known that TFs are usually involved in the regulation of important functional genes to help cells defend against abiotic stresses. However, the correlations between TFs and astaxanthin synthesis have never been reported. Based on the transcriptome data, 27 astaxanthin synthesis-related genes were targeted to six TFs. Hence, both DETFs and astaxanthin synthesis-related genes were taken into consideration with their targeting information to generate a valuable regulatory network (Figure 9). According to the regulatory network, six important TFs were found, suggesting that they might be involved in regulating 27 astaxanthin synthesis-related genes. Those TFs belong to MYB, MYB_related, NF-YC, Nin-like, and C3H families, among which there were four upregulated genes and two downregulated genes. Four TFs, including MYB, Nin-like, and C3H, were positive to *CrtO*, whereas two TFs such as MYB_related and NF-YC were negative to *CrtO*. Moreover, all of them could affect other astaxanthin synthesis-related genes by directly regulating *CrtO*, showing that *CrtO* was the hub gene of astaxanthin synthesis. NF-YC could also regulate the expression of CCDs. Furthermore, astaxanthin synthesis-related genes also existed in mutual modulation. For example, most of the astaxanthin synthesis-related genes were positive to each other. Nevertheless, both ZEP and CYP97C1 were negative to *CrtO* and CCDs (Figure 9).

## Discussion

*Haematococcus pluvialis* always attracts researchers because of its special ability to accumulate so much astaxanthin in many stress conditions such as high light and high salt (Domínguez-Bocanegra et al., 2004; He et al., 2007; Raman et al., 2008; Lemoine and Schoefs, 2010; Su et al., 2014). Meanwhile, mining genes related with astaxanthin synthesis has succeeded helping us to clarify the synthetic pathway of astaxanthin in *H. pluviali*s (Steinbrenner and Linden, 2001; Lemoine and Schoefs, 2010; Uri et al., 2019). The secrets why *H. pluvialis* can accumulate so much astaxanthin are still unknown, urging us to study more, including transcriptome analysis. All results indicated that improving the transcription level of astaxanthin synthesis-related genes leads to a significant increase of astaxanthin products when treated with many kinds of stresses (Huang et al., 2006; Raman et al., 2008; Gao et al., 2012b; He et al., 2018; Zhao et al., 2020). The regulation of astaxanthin synthesis would happen at the transcriptional level. However, the regulatory mechanism of astaxanthin synthesis at the transcriptional level is still kept unclear, hindering us from further improving astaxanthin production. In recent years, promoters of astaxanthin synthesis-related genes such as *bkt*, *crtO*, and *crtR-B* were isolated, finding that the promoters owned plenty of *cis*-elements including G-box, I-box, AE-box, MBS, and so on (Meng et al., 2005; Gao et al., 2010; Lu et al., 2010; Wang et al., 2012), and could respond to high light and sodium acetate (Wang et al., 2016). However, it is a pity that the research on TFs binding to these *cis*-elements made little progress.

To construct the regulatory network including astaxanthin synthesis-related genes and TFs, we tried to obtain dataset and expression trends of TFs and astaxanthin synthesis-related genes from the *H. pluvialis* transcriptome data treated with HLS stress for 1.5 h. Many research showed that the transcriptional of astaxanthin synthesis-related genes and astaxanthin production kept increasing under many kinds of stresses (Raman et al., 2008; Gao et al., 2012a; Wen et al., 2015). In these cases, the transcription of astaxanthin synthesis-related genes such as *psy*, *pds*, *bkt*, and *chy* could significantly increase after stressed for 4–8 h (Steinbrenner and Linden, 2001, 2003; Gao et al., 2012a). In this research, the transcription of astaxanthin synthesis-key genes such as *bkt* and *crtR-B* could be observed after stress for 0.5 h and notably increased after 2.5 h, although astaxanthin accumulation was detected at 4 h. Hence, we believed that their transcriptional regulation would have happened in an early stage of stress. Finally, 476 TFs belonging to 59 TF families were found in the transcriptome data of *H. pluvialis* under HLS stress for 1.5 h, indicating that a large number of TFs involved in the transcriptional regulation and stress response at an early stage of stress. This is the first report providing the largest TFs in the amount and variety in *H. pluvialis*.

Transcriptome data of our research found a great number of genes involving in cell process, metabolic process, single-organism process, catalytic activity, and binding, with active metabolite synthesis and inactive protein synthesis. DEGs mainly enriched in metabolic process, catalytic activity, single-organism process, single-organism cellular process, and single-organism metabolic process, which is similar to previous reports (Gao et al., 2012a, 2015; Li et al., 2017; He et al., 2018). It was no doubt that the most concerned synthetic pathway was carotenoid synthesis, and results showed that most of the upregulated DEGs were involved in lipid substances and carotenoid biosynthesis, whereas the downregulated DEGs were significantly enriched in DNA replication and photosynthesis. Moreover, our results provided more details indicating that astaxanthin synthesis-related genes were distributed into three expression trends with the transcription peak at 1.5–3, 24–48 h, or two peaks at 1.5–3 and 24–48 h. To understand the regulatory mechanism of astaxanthin synthesis in *H. pluvialis*, we should pay more attention to the time point of 1.5–3 and 24–48 h, which would be the important time point for regulation under HLS stress. Although so much transcriptome data were reported to study the correlation between astaxanthin synthesis and fatty acid synthesis and the transcriptional profiles of genes associated with them (He et al., 2018), little research on the transcription regulation was reported, especially the important TFs. Indeed, only 59 TFs could not support systematic analysis on the correlation between TFs and astaxanthin synthesis, which lead to no report until now (Gao et al., 2015). As we knew, TFs play important roles in responding to environmental stresses so that their expression is also affected by stresses. Previous research showed that *H. pluvialis* could respond to different environmental stresses by increasing the transcription activity of astaxanthin synthesis-related genes (Huang et al., 2006; Raman et al., 2008). As showed in our results, the transcription activity of TFs such as C2H2, COLIKE, G2like, ORPHANS, and PHD significantly improved under HL or HS. Interestingly, our results also indicated that TFs showed very different transcription profiles under HL or HS, although they were from the same TF families. For example, four TFs from ORPHANS showed very different transcriptional profiles, in which ORPHANS-1 was positive to HL, ORPHANS-2 was normal sensitive to HL and HS, and ORPHANS-3 was positive to HS, whereas the ORPHANS was negative to HL and HS. These phenomena might explain why *H. pluvialis* quickly responded to different environmental stresses. In addition, the TFs found in this research have not been reported elsewhere. So, further clarifying their functions would surely contribute to the study on the stress response mechanism of *H. pluvialis.*

In the present study, it was gratifying that up to 476 TFs and 79 DETFs were found from the *H. pluvialis* at the early stage of stress, providing the possibility to explore the correlation between TFs and astaxanthin synthesis. By constructing co-expression regulatory networks, we could find the important functional genes and identify hub genes in expression regulatory networks (Hollender et al., 2014; Zhao et al., 2018). Here, the TF–astaxanthin synthesis regulatory network pointed out that TFs modulated astaxanthin synthesis at the transcriptional level by regulating the transcription of *CrtO*, indicating that *CrtO* was at the core of astaxanthin synthesis, which is evidenced by many references proposing that β-carotene ketolase was the key enzyme in astaxanthin synthesis (Lemoine and Schoefs, 2010; Uri et al., 2019). In addition, six important TFs were involved in regulating astaxanthin synthesis-related genes, among which four TFs were positive, whereas two TFs were negative, showing that their regulation was multidimensional at the transcriptional level. Research on plant TFs confirmed that MYB TFs involved in plant stress resistance and flavonoid synthesis could bind to the MBS *cis*-element in the promoter, which was found in astaxanthin synthesis-related genes with high frequency (Meng et al., 2005; Gao et al., 2010; Wang et al., 2012). Also, the TFs of NF-YC, Nin-like, and C3H were also involved in drought stress and salt stress. Interestingly, we noticed that ZEP and CYP97C were negative to astaxanthin synthesis by affecting *CrtO* and CCDs. ZEP could convert zeaxanthin to violaxanthin, which would further produce abscisate so that downregulation of ZEP was observed in many kinds of abiotic stresses (Gao et al., 2015; He et al., 2018). Therefore, inhibiting ZEP expression might improve astaxanthin production. However, CYP97C1 was reported to be upregulated under high light and downregulated under SA and JA stresses (Gao et al., 2015; He et al., 2018). So, further researches might conduct to classify the role of CYP97C1. Hence, our findings provide a new clue on the astaxanthin synthesis.

The present studies provided the TF dataset of *H. pluvialis*, helping us to make clear the TF function in responding to environmental stresses. By constructing the TF–astaxanthin synthesis regulatory network, we could obtain their correlations and find important TFs involved in the transcription regulation under HLS stress, providing a theoretical foundation for artificial control of astaxanthin synthesis.

## Conclusion

In conclusion, our transcriptome data found 83,869 unigenes, among which there were 4,367 DEGs composed of 2,050 upregulated unigenes and 2,317 downregulated unigenes in *H. pluvialis* under HLS stress for 1.5 h. Most of DEGs were involved in metabolic process, catalytic activity, single-organism process, single-organism cellular process, and single-organism metabolic process at the early stage of stress. In addition, 476 TFs belonging to 52 families were identified and 69 DETFs including 28 upregulated TFs and 41 downregulated TFs. The TFs such as C2H2, COLIKE, and others showed different transcription profiles under HL or HS during 6 h, showing that they were involved in response to the environmental stresses during the early stage of stress. Finally, there were six TFs belonging to MYB, MYB_related, NF-YC, Nin-like, and C3H families that might be involved in the transcription regulation of 27 astaxanthin synthesis-related genes. Moreover, four TFs, including MYB, Nin-like, and C3H were positive to *CrtO*, whereas two TFs such as MYB_related and NF-YC were negative to *CrtO*. In brief, these TFs could affect astaxanthin synthesis by directly regulating *CrtO*. The present study provides new insight into the global view of TFs and their correlations with astaxanthin synthesis in *H. pluvialis*.

## Data Availability Statement

The data presented in the study are deposited in the NCBI repository, accession number SRR6816386.

## Author Contributions

KW and JN performed the experiments. KW, JN, QL, and CW collected the data and carried out all the analyses. CW and YY initiated the preparation of the manuscript. CW and HL conceptualized the idea and revised the manuscript. All authors have read and approved the final manuscript.

## Conflict of Interest

The authors declare that the research was conducted in the absence of any commercial or financial relationships that could be construed as a potential conflict of interest.

## Publisher’s Note

All claims expressed in this article are solely those of the authors and do not necessarily represent those of their affiliated organizations, or those of the publisher, the editors and the reviewers. Any product that may be evaluated in this article, or claim that may be made by its manufacturer, is not guaranteed or endorsed by the publisher.

## Footnotes

1
http://planttfdb.cbi.pku.edu.cn/
