# Supplementary material for: Transcription Factors From Haematococcus pluvialis Involved in the Regulation of Astaxanthin Biosynthesis Under High Light-Sodium Acetate Stress
Source: Front Bioeng Biotechnol. 2021 Oct 25;9:650178. doi: 10.3389/fbioe.2021.650178 (PMC8573195; doi:10.3389/fbioe.2021.650178)
Supplement: Supplementary file 1 [file Data_Sheet_1.docx]

# Supplementary Figures and Tables

TABLE S1 List of the primers

| Primer name | seqence(5'-3') |
| --- | --- |
| bHLH-F1 | TGCTGTCAGGAGCTGGGATC |
| bHLH-R1 | AGGGTGCTGAAGGCGTGGTG |
| bZIP-F1 | CAGTACCGCAAGTCCCTTTG |
| bZIP-R1 | TCTGTCGCGTCCTCTTCCTC |
| C2C2-CO-like-F1 | GCTCCTCCGAGGTTCACTCA |
| C2C2-CO-like-R1 | GAAGGCATTTGCGGTACAAT |
| C2C2-GATA1-F1 | TCGGGTTTGTGGGTTGTGAA |
| C2C2-GATA1-R1 | ACCAGTGGCACTCCCTCCTC |
| C2C2-GATA2-F1 | AAAGCAGCGAGGATGGATAT |
| C2C2-GATA2-R1 | AGCAACAAGGCTCTACAGGA |
| C2H2-F1 | GCTCCCGCGTGTTCTGCTTA |
| C2H2-R1 | GGCTTCTTGTGGACCTTGTT |
| CCAAT1-F1 | AAGCGACCCAGCGGAGTTCA |
| CCAAT1-R1 | GCAGAGTGCGCCTCTTATTC |
| CCAAT2-F1 | AGACAGCCTTCACTTACGCC |
| CCAAT2-R1 | CGAATGGGATGATGTTCTGG |
| CPP-F1 | GACAGGGGTGTTCTGCGATG |
| CPP-R1 | GAGGGTTGTTCATGCACGTG |
| G2-like-F1 | AGCTGATGGGCGTAGATGGT |
| G2-like-R1 | CCCGCAACTGTGGTGTAGAT |
| GNAT1-F1 | GTGGTGCTGGAGGCAGAGGT |
| GNAT1-R1 | AGCTGGTTGAGTGAGGGATC |
| GNAT2-F1 | GCGTGGTCTTTCCGCTCAAG |
| GNAT2-R1 | ATGCCATAGCCCCTGTAGGG |
| GNAT3-F1 | GGATGGGACGAATGTCAATG |
| GNAT3-R1 | CTTGTTCTTGCGGTTGTTGA |
| GNAT4-F1 | GGTGGAGTTGCCCGTGAATG |
| GNAT4-R1 | CGTGGAGGAGGGCTATGTCG |
| HB-F1 | AGCAGAAGCCTTTGAACAGA |
| HB-R1 | TGGAGTACAGGGCCAAGATA |
| MBF1-F1 | ATTCAATGCGGGCACTAACA |
| MBF1-R1 | CATACTCCTGGATGATTTGG |
| MYB1-F1 | TTAGTGGGTGTTCGCCAAGC |
| MYB1-R1 | ATCCCGCGAGTAGAGGTTCA |
| MYB2-F1 | GACTATTCCGAATGCCTTTA |
| MYB2-R1 | AACTGAGACAAGCCAATCAA |
| Orphans1-F1 | TATGAACATGGGCGACAACG |
| Orphans1-R1 | CTGCTGAATGCTTGCGGTAC |
| Orphans2-F1 | CGCCTCCCGCATGTTTATGT |
| Orphans2-R1 | CTTGCCACTGCTGCTGACCC |
| Orphans3-F1 | GAGGATGGCGATGGCGTTGT |
| Orphans3-R1 | GGCTTGCGGCTGGTAGAGGT |
| Orphans4-F1 | CGGTATGGGACTGTGATGGT |
| Orphans4-R1 | GACGCAAAGTGTTGAAGGAT |
| PHD-F1 | ATGAACAGGTACGCAAAGGT |
| PHD-R1 | TCGTCGTTGTCACATCCCAC |
| RWP-RK1-F1 | CAAGCGGACTGTGGACTGGG |
| RWP-RK1-R1 | TGTCAGGTCGGGCTGCGTCT |
| RWP-RK2-F1 | AGCCCAACGACTACACCCTC |
| RWP-RK2-R1 | ACTTGCTCCGCCTGCTCTAA |
| SNF2-F1 | CCGTGTTCCTGTTCCTGCTT |
| SNF2-R1 | CGATAGTGCCGCTGGTGATG |
| TRAF1-F1 | GCCGAGGATGCGGAGAATGA |
| TRAF1-R1 | GCCCAGGAAGACGGCACAGA |
| TRAF2-F1 | GCGGACTTTGAAATGGTGGT |
| TRAF2-R1 | CTCAGGGAGGTTGTCGGTGT |
|  |  |
| AP2-EREBP-F1 | TGTTGCACTGGGCTCCTCAC |
| AP2-EREBP-R1 | CGCTGTCTCACGCCTCTGTA |
| C2C2-GATA-F1 | CCCTTGGCCTGTCTCCGTAC |
| C2C2-GATA-R1 | TGCCTGGAAGCTCAACTCCT |
| C3H-F1 | TGATTGCTATGAACGAGGCG |
| C3H-R1 | GTGGCTGTTGCGGCATTTGT |
| CCAAT-F1 | AACCAAACAGCCAGCCTTTA |
| CCAAT-R1 | GCACCAGCTCTTCAAACTCC |
| Co2-F1 | AGGACGCTGAGCTGAAACCC |
| Co2-R1 | CGCCATCCTGCTCGTAGAAC |
| CSD-F1 | CTGGAGTCAAGGTGCGTCTG |
| CSD-R1 | GCAGGTAGTCCACCATCTCA |
| E2F-DP-F1 | TTGAAGCAGTACGGCCAAAT |
| E2F-DP-R1 | GATGCCCAAAGCCAACAGGA |
| MED7-F1 | CACCAGAAGCTCCACTTTGC |
| MED7-R1 | ACTCCATTTCAGCCATACGC |
| MYBa-F1 | AGTGCAGGCGTCACCAGTCA |
| MYBa-R1 | CCCTAGCTCCTTGCCAAACT |
| MYBb-F1 | CCCCATCCAATCCCTTCCTG |
| MYBb-R1 | TGTGCGTCGGTGGCCTTCAT |
| MYBc-F1 | GAAGAAGGGCGACTGGGATG |
| MYBc-R1 | GAGGAGGTATGCGAGCAAGG |
| Orphans-F1 | GGCAATAGACCTGGTGGATG |
| Orphans-R1 | GGAAGCGAAAGTCACGGTAG |
| PHD-F1 | CGGCCATACAGTGGGTTGCA |
| PHD-R1 | ACATGCCCTTCCGTGATGAA |
| SETa-F1 | GAGCAGTTGGTGGTGAGGGC |
| SETa-R1 | TTGGGTAAGCTGGGTGGGTC |
| SETb-F1 | CGACGAATTGAGTGGGAGTG |
| SETb-R1 | CTCGCACAAACGCTTGGACT |
| SETc-F1 | GCATGGGTCATAGCTCACGG |
| SETc-R1 | TTGAAGCGACTGTCTGGGTG |
| SETd-F1 | GTGATGGGCAGCCTGGGTCA |
| SETd-R1 | CTCGCAACGCCTTCTTCTCG |
| SETe-F1 | TGCAGGTGGTTGTACGATTT |
| SETe-R1 | TTCACAGTTGGGTTGGAAGG |
| SNF2a-F1 | AGGTGCTGGTGCTGCTGGTG |
| SNF2a-R1 | GCTGCCCTTGATGGTGATGC |
| SNF2b-F1 | CATCTGGCTCTTCCTCCTCT |
| SNF2b-R1 | CAGCATCTTGCCCTCTACAC |
| SNF2c-F1 | TATCGAGACGGTCAGAAGCG |
| SNF2c-R1 | CGTGTTCAATGAAAGCGTGA |
| SNF2d-F1 | GCAACCAAGAAGAAAGGGAC |
| SNF2d-R1 | CTCACGCACCACCTTACTGA |
| WRKY-F1 | GCAAACGACGACGGCTATCA |
| WRKY-R1 | AGCAGGAGCTGGGTGGGTGT |
| SETa2-F1 | GCTGCCAGACAGTGCCTTGA |
| SETa2-R1 | GACCTCCCGTATCAGCCCAC |
| Actin1 | AGCGGGAGATAGTGCGGGACA |
| Actin2 | ATGCCCACCGCCTCCATGC |

TABLE S2 List of the genes associated with astaxanthin synthesis

| **Gene ID** | **Annotation** | **Gene Name** |
| --- | --- | --- |
| Ch_GLEAN_10007057 | isopentenyl diphosphate isomerase | IPI |
| XLOC_013259 | isopentenyl diphosphate isomerase |  |
| XLOC_051007 | isopentenyl pyrophosphate isomerase |  |
| Ch_GLEAN_10010046 | chloroplast geranylgeranyl diphosphate synthase | GGPS |
| XLOC_048896 | chloroplast geranylgeranyl diphosphate synthase |  |
| XLOC_048897 | chloroplast geranylgeranyl diphosphate synthase |  |
| XLOC_053142 | chloroplast geranylgeranyl diphosphate synthase |  |
| XLOC_042543 | phytoene synthase | PSY |
| XLOC_048563 | phytoene synthase |  |
| Ch_GLEAN_10011505 | phytoene desaturase | PDS |
| XLOC_006571 | phytoene desaturase |  |
| Ch_GLEAN_10009908 | chloroplast zeta-carotene desaturase | ZDS |
| XLOC_015032 | chloroplast zeta-carotene desaturase |  |
| XLOC_030044 | chloroplast zeta-carotene desaturase |  |
| Ch_GLEAN_10007045 | lycopene beta cyclase | LCY |
| Ch_GLEAN_10010036 | lycopene beta cyclase |  |
| Ch_GLEAN_10010071 | lycopene beta cyclase |  |
| Ch_GLEAN_10009207 | beta-carotene ketolase | BKT |
| Ch_GLEAN_10009310 | beta-carotene ketolase |  |
| XLOC_038901 | beta-carotene ketolase |  |
| XLOC_039544 | beta-carotene ketolase |  |
| Ch_GLEAN_10005232 | carotenoid hydroxylase | CRTZ |
| Ch_GLEAN_10006000 | beta-carotene hydroxylase | crtR-B |
| Ch_GLEAN_10011708 | beta-carotene hydroxylase |  |

TABLE S3 The expression of astaxanthin synthesis- related genes from 0h to 48h

| **Gene ID** | **1.5h** | **3h** | **6h** | **9h** | **12h** | **24h** | **48h** |
| --- | --- | --- | --- | --- | --- | --- | --- |
| Ch_GLEAN_10010071 | 2.41 | 2.60 | 1.80 | 1.33 | 1.21 | 1.65 | 2.07 |
| XLOC_013259 | 0.67 | 0.81 | 0.78 | 0.74 | 0.76 | 0.88 | 0.87 |
| XLOC_048563 | 3.98 | 6.50 | 3.08 | 2.65 | 2.66 | 5.13 | 6.33 |
| XLOC_051007 | 1.65 | 2.13 | 1.66 | 1.70 | 1.73 | 1.78 | 2.03 |
| XLOC_015032 | 1.60 | 1.98 | 1.01 | 0.77 | 0.69 | 1.68 | 1.89 |
| Ch_GLEAN_10010036 | 2.83 | 3.38 | 1.96 | 1.48 | 1.40 | 2.25 | 2.16 |
| Ch_GLEAN_10011505 | 2.54 | 3.01 | 1.72 | 1.49 | 1.38 | 1.86 | 1.67 |
| Ch_GLEAN_10005232 | 1.87 | 8.10 | 4.84 | 4.42 | 4.99 | 16.07 | 16.03 |
| XLOC_048897 | 0.75 | 1.22 | 1.11 | 1.07 | 0.94 | 1.58 | 1.34 |
| XLOC_039544 | 3.82 | 3.38 | 1.43 | 1.28 | 1.29 | 1.42 | 2.09 |
| XLOC_042543 | 3.60 | 5.82 | 2.78 | 2.26 | 2.57 | 4.52 | 5.52 |
| XLOC_030044 | 1.49 | 1.91 | 1.10 | 0.86 | 0.78 | 1.65 | 1.77 |
| Ch_GLEAN_10007057 | 0.74 | 0.91 | 0.85 | 0.75 | 0.78 | 1.04 | 1.01 |
| XLOC_048896 | 0.62 | 1.13 | 0.98 | 1.02 | 0.90 | 1.27 | 1.33 |
| Ch_GLEAN_10009310 | 1.62 | 1.73 | 1.21 | 1.13 | 1.27 | 1.17 | 1.27 |
| XLOC_038901 | 6.51 | 12.93 | 12.25 | 10.46 | 9.31 | 19.60 | 14.76 |
| Ch_GLEAN_10007045 | 3.64 | 3.57 | 2.27 | 1.86 | 1.67 | 2.70 | 2.65 |
| Ch_GLEAN_10009207 | 4.24 | 4.00 | 1.48 | 1.20 | 1.44 | 2.02 | 2.66 |
| Ch_GLEAN_10006000 | 23.86 | 18.45 | 6.59 | 4.45 | 3.51 | 3.20 | 6.42 |
| Ch_GLEAN_10011708 | 57.36 | 47.24 | 17.31 | 10.15 | 8.45 | 7.64 | 13.96 |
| Ch_GLEAN_10009908 | 1.34 | 1.53 | 0.79 | 0.53 | 0.60 | 1.34 | 1.41 |
| Ch_GLEAN_10010046 | 0.74 | 1.16 | 1.08 | 0.96 | 0.98 | 1.42 | 1.38 |
| XLOC_006571 | 2.68 | 3.35 | 1.83 | 1.51 | 1.40 | 2.09 | 1.93 |
| XLOC_053142 | 0.69 | 1.06 | 0.96 | 0.90 | 1.00 | 1.61 | 1.56 |

**
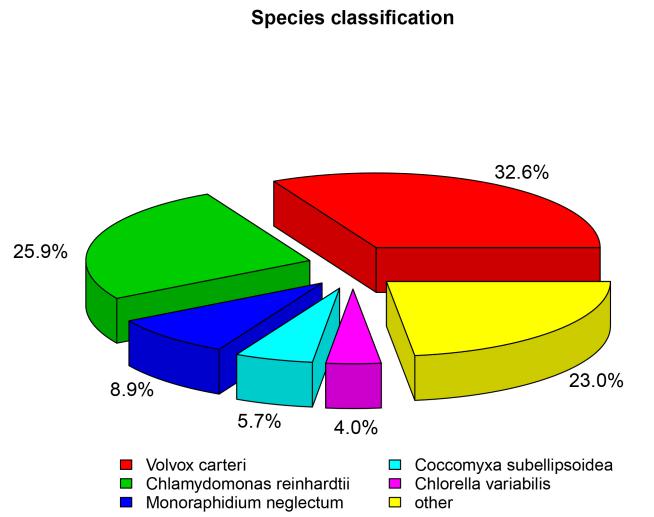
**

FIGURE S1 Homology analysis of *H. plusvialis*.


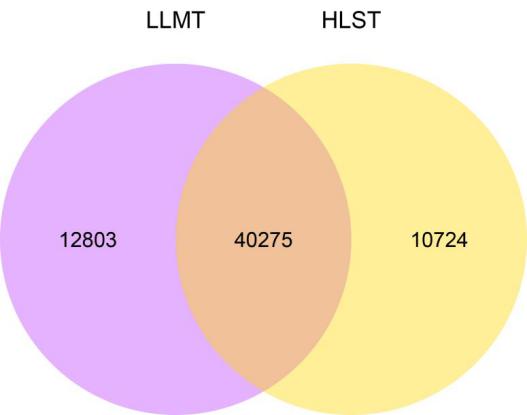


FIGURE S2 Differentially expression genes in Venn diagram.


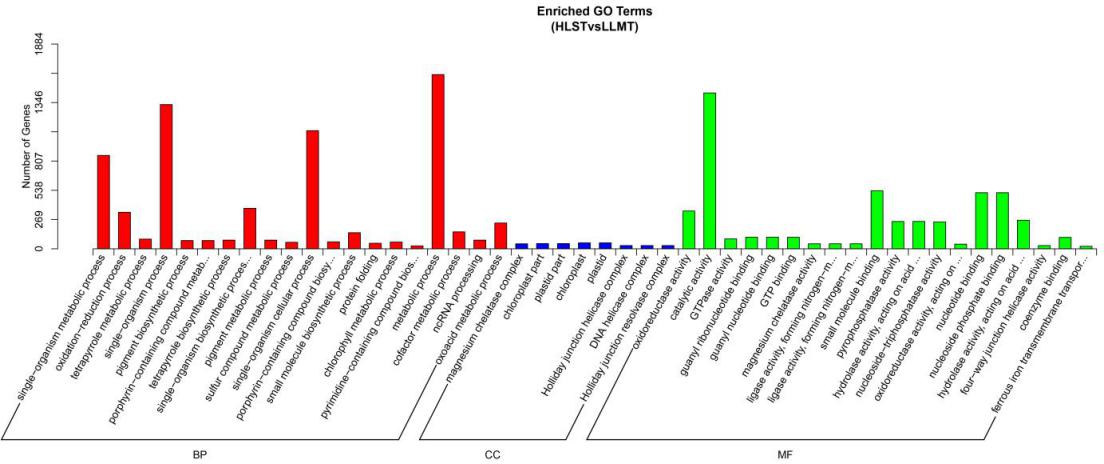


FIGURE S3 Gene ontology enrichment of DEGs
